# MicroRNA-875-5p inhibits the growth and metastasis of cervical cancer cells by promoting autophagy and apoptosis and inhibiting the epithelial-mesenchymal transition

**DOI:** 10.3389/fonc.2024.1361721

**Published:** 2024-05-10

**Authors:** Yingxiu Liang, Chunyang Li, Xiaohong Hou, Yiguang Lin, Jing Cheng

**Affiliations:** ^1^ Reproductive Center, Department of Obstetrics and Gynecology, The Second Affiliated Hospital and the Yuying Children’s Hospital of Wenzhou Medical University, Wenzhou, China; ^2^ Department of Biochemistry, School of Basic Sciences, Wenzhou Medical University, Wenzhou, China; ^3^ School of Pharmaceutical Science, Guangdong Pharmaceutical University, Guangzhou, China; ^4^ Department of Traditional Chinese Medicine (TCM), Third Affiliated Hospital of Sun Yat-Sen University, Guangzhou, China; ^5^ Research & Development Division, Guangzhou Anjie Biomedical Technology Co. Ltd, Guangzhou, China

**Keywords:** microRNA, cervical cancer, autophagy, apoptosis, tumor biomarker, tumorigenesis, autophagy, apoptosis

## Abstract

**Introduction:**

MicroRNA-875-5p (miR-875-5p) is a cancer-related microRNA. It has been demonstrated that miR−875−5p participates in the development of various types of cancer such as hepatocellular carcinoma, gastric carcinoma, prostate and bladder cancer. Previous research suggested that miR-875 is implicated in the development of cervical cancer cells. However, the exact role and function of miR−875−5p in cervical cancer remain unexplored. It is important to examine the role and function of miR-875-5p and the associated signaling pathway, as the findings may have diagnostic and therapeutic significance. Thus, in this study, we investigated the effect of miR-875-5p on the growth and metastasis of cervical cancer cells and the possible underlying mechanisms.

**Methods:**

Reverse transcription-quantitative polymerase chain reaction (RT-qPCR) was used to detect the expression of miR-875-5p in cervical cancer cells and normal cervical epithelium. After overexpression or co-expression of miR-875-5p in cells, the changes in cell function were analyzed. Western blot was used to detect the expression changes of epithelial-mesenchymal transition (EMT) -related proteins and autophagy-related proteins.

**Results:**

Functional studies demonstrated that miR-875-5p overexpression significantly inhibited the proliferation, migration, invasion, and EMT, and promotes apoptosis and autophagy of cervical cancer cells., while miR-875-5p knockdown promoted the proliferation, migration, invasion, and EMT, and inhibited apoptosis and autophagy cervical cancer cells. Furthermore, Western blot results showed that overexpression of miR-875-5p downregulated the expressions of N-cadherin, Snail, Vimentin and microtubule-associated protein 1 light chain 3B I (LC3B I). Conversely, miR-875-5p upregulated the expression of E-cadherin.

**Conclusion:**

In conclusion, our findings suggest that miR-875-5p functions as a tumor inhibitor suppressing the growth and metastasis of cervical cancer. Overexpression of miR-875-5p inhibits malignant behavior and promotes autophagy and apoptosis in cervical cancer cells. These findings advance our understanding of the role and function of miR-875-5p in cervical cancer and could facilitate the development of early genetic markers or biomarkers and therapeutic targets for cervical cancer.

## 1 Introduction

Cervical cancer (CC) is the fourth most common cancer among women worldwide, posing a serious threat to women’s health (1). Each year, approximately 604,000 women are diagnosed with CC and over 342,000 deaths worldwide due to CC (2). The carcinogenesis of CC, from precancerous lesion to cancer, is a complex process. Currently, persistent viral infection with the high-risk human papilloma virus (HPV) has been identified as the predominant cause of the onset of CC (3). However, there is increasing evidence to support that HPV infection alone is insufficient to fully explain the initiation and development of all CCs (4). At present, the main preventions for CC include HPV screening and prophylactic HPV vaccines. While the three types of prophylactic vaccines on the market are effective in preventing HPV infections, they provide limited benefits to women with pre-existing infections (5). The main treatments for CC include surgery, radiotherapy, chemotherapy and newer therapies including immunotherapies, targeted therapies, and genetic modification treatment approaches (6–11).. Standard treatment for locally advanced CC, pelvic radiation therapy combined with cisplatin, failed in approximately one third of patients and has not improved in nearly 20 years. Furthermore, the efficacy of treatments for recurrent and metastatic CC is limited (6). Therefore, there is an urgent need to search for more effective therapies by identifying new molecular targets for CC.

MicroRNAs (miRNAs/miRs) are a class of small endogenous noncoding RNAs that regulate gene expression by binding to the 3’untranslated region (3’-UTR) of target mRNAs and are involved in the regulation of various biological processes, including inflammation, cell growth, apoptosis, invasion and migration (12). For instance, one study showed that miR-215-5p were highly expressed in inflammatory tissues, might promote the expression levels of pro-inflammatory cytokines including IL-1β and IL-22, with potential regulation of chronic inflammation and colon carcinogenesis chronic (13). Ectopic expression of miR-424 in precursor cells enhances monocytic differentiation (14). Another study suggested that the IL-11/IL-11R signaling pathway in monocytes was a therapeutic target for relapsing-remitting multiple sclerosis (15). Moreover, overexpression of miR-223 significantly increased the number of granulocytes (14). The immune microenvironment and cervical tumor glycolysis are interconnected (6). Furthermore, abnormal change in specific miRNA expression is associated with cancer progression (16). Muhammad et al. found that miR-203 was highly upregulated in breast cancer tissues, anti-miR-203 suppresses breast cancer cell proliferation *in vitro (* 17). The expression of miR-17-92 is elevated in hepatocellular carcinoma (HCC) and is associated with a poor prognosis. Overexpression of miR-17-92 in HCC cells significantly accelerated the proliferation and migration of HCC cells (18). The miR-766-3p inhibited the progression of gastric cancer by regulating the target gene and regulating the signal pathway (19). In addition, accumulating evidence suggests that dysregulated miRNAs play a crucial role in CC. MicroRNA-144 acts as a tumor suppressor in the proliferation and metastasis of CC cells by directly targeting vascular endothelial growth factor A and vascular endothelial growth factor C (4). Furthermore, miR-92a-3p promotes proliferation, invasion, and cell cycle transition in CC stem cells by targeting the large tumor suppressor 1 (LATS1) (20). miR-5003-3p has been shown to promote the epithelial-mesenchymal transition (EMT) in CC by directly targeting osteoglycin (16).

MicroRNA-875-5p, originally identified as a novel therapeutic target for prostate cancer, was shown to counteract the epithelial-to-mesenchymal transition and enhance the radiotherapy response (21). Currently, numerous studies have also shown that miR-875-5p acts as a tumor suppressor in different types of human cancer, including HCC (22), gastric carcinoma (23), prostate cancer (21), and bladder cancer (24). A recent study reported that miR-875-5p inhibited the proliferation, migration and invasion of gastric cancer cells *in vitro* and inhibited tumorigenesis *in vivo* (25). Furthermore, miR-875 is associated with the occurrence and development of CC (26–28). A study by Lin et al. showed that miR-875 inhibits the expression of both the synthetically exogenous E6 and the endogenous E6 oncogene, which are the main drivers of the oncogenic cascade in the cervical epithelium (26, 27). A high level of miR-875 can inhibit cell growth and promote apoptosis in HPV16 positive CC cells (26).

Despite miRNAs have been shown to be involved in the initiation and development of various cancers, no studies have been conducted to investigate the functional role and related pathways of miR-875-5p in CC. However, studies of this kind can reveal novel biomarkers or therapeutic targets. Therefore, in the present study, we investigated the expression of miR-875-5p in CC cells and explored its potential effects on proliferation, apoptosis, migration and invasion of CC cells. Furthermore, the study evaluated the effects of miR-875-5p on cell autophagy and EMT. The findings of the study would advance our understanding of the role of miR-875-5p in CC.

## 2 Materials and methods

### 2.1 Cell culture

Human normal cervical epithelial cells, C33A, Caski, HeLa, MS751 and SiHa cervical cancer cell lines were obtained from the American Type Culture Collection (ATCC, Manassas, VA, USA). Cells were grown at Roswell Park Memorial Institute 1640 (RPMI-1640, Gibco, Grand Island, NY, US) supplemented with 10% fetal bovine serum (FBS, Gibco, Grand Island, NY, US) and 1% penicillin/streptomycin (Gibco, Grand Island, NY, US) in a humidified incubator with 5% CO2 at 37°C. Mycoplasma was routinely checked in the research laboratory on a monthly basis. Cell lines used were authenticated by short tandem repeat (STR) analyses. Cells in the logarithmic growth phase were used for further study.

### 2.2 Oligonucleotide transfection

The miR-875-5p mimic (5’−CACCTGATAACTGAGGTATA−3’), mimic control (5’−TATACCTCAGTTTTATCAGGTG−3’), inhibitor (5’−CACCTGATAAAGTGAGGTATA−3’), and inhibitor control (5’−CAGTACTTTTGTGTAGTACAA−3’) were chemically synthesized and purchased from Shanghai HANBIO (Shanghai, China) and transfected into CC cells according to the instruction provided by the manufacturer. After 6 h of transfection, the medium was replaced with RPMI-1640 containing 10% FBS and 1% penicillin/streptomycin. Cells were harvested 48 h later.

### 2.3 Reverse transcriptase−quantitative polymerase chain reaction analysis assay

The expression of miR-875-5p in CC cell lines was detected by RT-qPCR assay. Briefly, total RNA was extracted from cells using TRIzol reagent (ThermoFisher, Waltham, MA, USA), miRNA expression levels were quantified using a TaqMan miRNA real-time RT-PCR kit (ThermoFisher, Waltham, MA, USA) according to the manufacturer’s instructions. Data analysis adopts Applied Biosystems 7500 Real Time PCR. Universal small nuclear RNA U6 (RNU6b) and glyceraldehyde-3-phosphate dehydrogenase (GAPDH) were used as endogenous controls for miRNAs. Each sample was examined in triplicate.

### 2.4 The Cell Counting Kit-8 assay

The viable cell mass was measured with the Cell Counting Kit-8 (CCK8) Assay (HANBIO, Shanghai, China). Cervical cancer cells in the logarithmic growth phase after transfection were seeded in 96-well plates at a density of 1× 10^4 cells/well with 100 μL culture system. The cells were then cultured in an incubator with 5% CO2 at 37°C for 24, 48, 72 and 96 hours respectively. A total of 10 μl CCK8 solution was added to each well and the cells were cultured for another 4 h. Finally, the absorbance was determined at 490 nm with a microplate reader. All procedures were carried out in biological triplicate.

### 2.5 Colony formation assay

Colony formation assay was used to evaluate the cell proliferation ability. Briefly, 5× 10^3 transfected CC cells were seeded in 10 cm dishes and cultured in RPMI 1640 medium for 10 days respectively. Each treatment should be carried out in triplicate. The colonies obtained were washed with PBS and fixed in 4% formalin (Sigma-Aldrich; Merck KGaA) for 10 min at room temperature and then washed with PBS followed by staining with 0.2% crystal violet (Sigma-Aldrich; Merck KGaA). All procedures were carried out in biological triplicate.

### 2.6 Flow cytometry assay

The flow cytometry assay was performed to evaluate apoptosis of CC cells. The transfected CC cells were seeded into 6-well plates and cultured in an incubator with 5% CO2 at 37°C for 48 h. Cells were then digested with trypsin, washed in PBS and resuspended to an adjusted cell suspension concentration of 2 × 10^4/ml, then fixed with precooled alcohol (75%). Totally 300 μl of propidium iodide was then added to the tube and the mixed solution was incubated for 15 min in darkness. The cell cycle was determined by flow cytometry. All procedures were carried out in triplicate.

### 2.7 Migration and invasion assay

A wound healing assay was performed to assess the migration ability of CC cells. Briefly, cells were seeded in 6-well plates at a density of 1 × 10^6 cells/well and cultured to a density of 90%. Cell monolayers were scraped with sterile 200µl pipette tips (EMD Millipore, Billerica, MA, USA) to generate scratch wounds and washed three times with PBS (Sigma-Aldrich; Merck KGaA) to remove cell debris and then immediately take an image under the microscope. Cells were incubated at 37°C with 5% CO2 for another 24 h and 48 h. To determine the migration distances, wounds were observed under an inverted microscope at x100 magnification and images were captured.

Another cell migration and invasion assay was performed using a Transwell chamber with or without polycarbonate membranes (8 μM pore size) (BD Biosciences, Franklin Lakes, NJ, USA). Briefly, 100 µl cell suspension was added to the upper chamber and 500 µl RPMI 1640 with 10% FBS as chemoattractant was added to the lower chambers. Cells were incubated at 37°C with 5% CO2 for 24 h. Following careful removal of the cells remaining on the upper surface of the membrane, those on the lower surface of the membrane were fixed in pure methanol (Sigma−Aldrich; Merck KGaA) for 20 min and stained with 0.1% crystal violet (Sigma-Aldrich; Merck KGaA) for 15 min. Stained cells were visualized and counted in five randomly selected fields under an inverted microscope at x200 magnification. All procedures were repeated over three times.

### 2.8 Western blotting

Western blotting was used to determine the levels of proteins. Briefly, cells were harvested with lysis buffer containing 1% protease inhibitor and phosphatase inhibitors. Total protein concentration was determined using an enhanced BCA protein assay kit (Beyotime Institute of Biotechnology, Haimen, China). Totally 40 μg of total protein was loaded onto a 10% SDS-PAGE gel, and after electrophoresis, the protein was transferred to polyvinylidene fluoride (PVDF) membranes, which was to be blocked with 5% defatted milk powder and incubated with primary antibodies of autophagy protein 5 (ATG5), microtubule-associated proteins 1 light chain 3B (LC3B), E-cadherin, N-cadherin, snail and vimentin at 4°C overnight. The PVDF membranes were then washed with TTBS buffer and probed with horseradish peroxidase (HRP)-conjugated secondary antibody (7,074/7,076; 1:2,000; Cell Signal Technology) at room temperature for 1 h. The immunosignal substance was detected with the SuperSignal West Dura Extended Duration Substrate Kit (Thermo Fisher Scientific), and the resulting images were captured with an imaging system (DNR BioImaging System, Jerusalem Israel). All procedures were repeated over three times.

### 2.9 Identification of target gene for miR-875-5p

To investigate the signaling pathway and the possible underlying mechanism by which miR−875−5p inhibits cervical cancer growth, candidate downstream targets of miR−875−5p were identified using bioinformatic tools (ENCORI/starBase). The ENCORI/starBase (https://rnasysu.com/encori/index.php) were used by entering ‘miR−875−5p’ into the search box to predict potential miRNA target genes and binding sites.

### 2.10 Statistical analysis

Statistical analysis was performed using GraphPad Prism 8.3.0 (GraphPad Software, CA, USA). Data are expressed as mean ± standard deviation (SD). Mean difference between the two groups was compared using the t-test, and the differences between multiple groups were compared using one-way ANOVA. The value of p < 0.05 is considered statistical significance.

## 3 Results

### 3.1 Expression levels of miR-875-5p in normal cervical epithelial cell line and several cervical cancer cell lines

**Figure 1:**
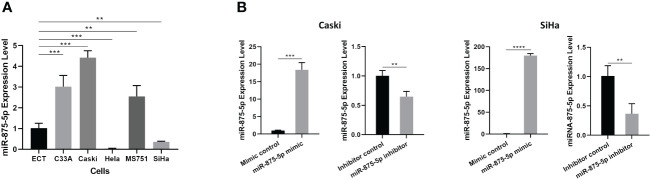
MicroRNA-875-5p was dysregulated in CC cells. **(A)** Expression of miR-875-5p in the normal cervical cell line ECT) and 5 CC cell lines (C33A, Caski, HeLa, MS751, and SiHa) determined by RT-qPCR. **(B)** Effect of miR-875-5p mimic control/mimic and miR-875-5p inhibitor control/inhibitor on its expression determined by RT-qPCR. **p < 0.01, ***p < 0.001, ****p < 0.0001.

The level of miR-875-5p in a normal cervical epithelial cell line and five CC cell lines (C33A, Caski, HeLa, MS751 and SiHa) was examined. **Figure 1A** demonstrated that in the five different CC cell lines, miR-875-5p levels were significantly higher in three CC cell lines (C33A, Caski, MS751) and significantly lower in two cancer cell lines (HeLa, SiHa) compared to normal cervical epithelial cell lines examined. Transfection of miR-875-5p mimic, mimic control, inhibitor, and inhibitor control was performed in SiHa and Caski cell lines, respectively. **Figure 1B** shows that miR-875-5p mimic transfection upregulated endogenous expression levels, while miR-875-5p inhibitor transfection downregulated expression in both Caski and SiHa cells.

### 3.2 Effects of miR-875-5p expression on proliferation in cervical cancer cells

**Figure 2:**
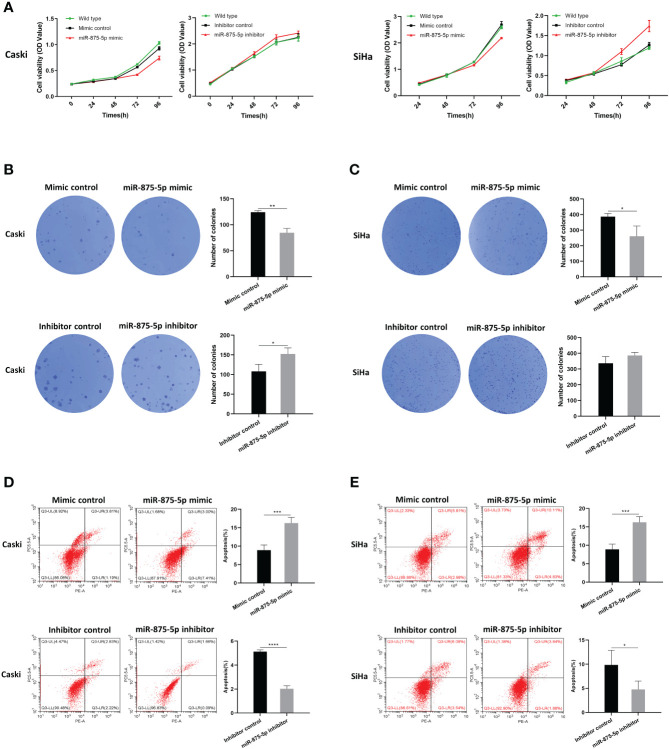
MicroRNA-875-5p inhibited the proliferation and promoted the apoptosis of CC *in vitro*. **(A)** CCK-8 assays detected changes in cell proliferation after overexpression or knockdown of miR-875-5p. **(B–C)** Colony formation assays detected cell proliferation ability after overexpression or knockdown of miR-875-5p. **(D–E)** flow cytometry evaluated cell apoptosis ability after overexpression or knockdown of miR-875-5p. *p < 0.05, **p < 0.01, ***p < 0.001, ****p < 0.0001.

The Cell Counting Kit-8 (CCK8) assay and colony formation assay were used to assess CC cell proliferation. As shown in **Figure 2A**, the results of the CCK8 assay showed that there were no significant differences in cell growth of SiHa and Caski cells after transfection of the miR-875-5p mimic. After transfection with the miR-875-5p inhibitor, there was also no significant change in cell growth. The results of the colony formation assay were presented in **Figures 2B**, **2C**. The results showed that overexpression of miR 875-5p decreased the growth rate of SiHa and Caski cells compared to the normal control, while inhibition of miR-875-5p only increased the growth of Caski cell lines. Low expression of miR-875-5p has no significant effect on the growth of the SiHa cell line.

### 3.3 Effects of miR-875-5p expression on apoptosis in cervical cancer cells

Flow cytometry was used to evaluate the role of miR-875-5p in CC cell apoptosis. As shown in **Figures 2D**, **2E**, the results of flow cytometry confirmed that overexpression of miR-875-5p can significantly increase apoptosis in SiHa cells and Caski cells, while miR-875-5p inhibition significantly inhibited apoptosis in two cell lines.

### 3.4 Effects of miR-875-5p expression on migration and invasion in cervical cancer cells

**Figure 3:**
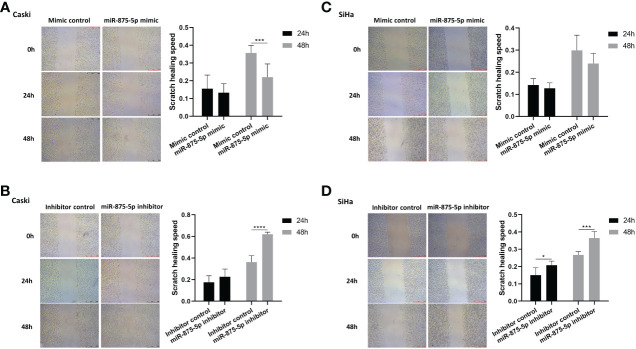
MicroRNA-875-5p inhibited the migration of CC *in vitro*. **(A–D)** Wound healing assays determinNAed the migration ability of cells after overexpression or knockdown of miR-875-5p. Scale bar, 100 μm. *p < 0.05, **p < 0.01, ***p < 0.001, ****p < 0.0001.

**Figure 4:**
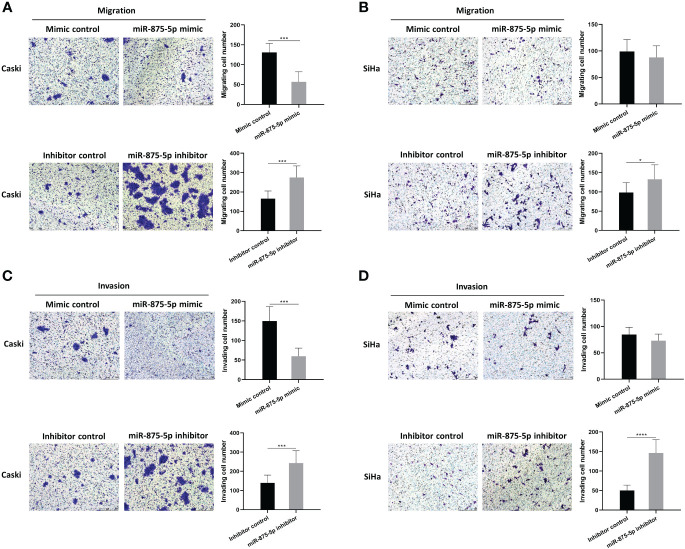
MicroRNA-875-5p inhibited the migration and invasion of CC *in vitro*. **(A–D)** Transwell assays determined the number of migrated CC cells and the cells invaded through the membrane in a Transwell system. Scale bar, 50 μm. *p < 0.05, ***p < 0.001, ****p < 0.0001.

The scratch wound healing assay and the transwell assay were used to assess the migration and invasion of CC cells. The results of the scratch wound healing assay showed that the overexpression of miR-875-5p decreased the healing speed of scratches in the Caski cell line, while it had no significant effect on the healing speed in the SiHa cell line (**Figures 3A, C**). The miR-875-5p knockdown increased the healing speed of scratches in the Caski and SiHa cell lines (**Figures 3B, D**). The result of the transwell migration assay showed that miR-875-5p mimic transfection significantly reduced the number of Caski cells that migrate to the lower level, but had no significant effect on the number of SiHa cell migration (**Figures 4A, B**). The number of cells that migrated to the lower chamber was significantly increased in Caski and SiHa cells transfected with miRNA inhibitors (**Figures 4A, B**). The result of the transwell invasion assay showed that transfection of miR-875-5p mimics significantly reduced the number of Caski cell invasion, but has no significant effect on the number of SiHa cells invasion (**Figures 4C, D**). There was a significant increase in the number of cells migration through the transwell membrane in Caski and SiHa cells transfected with miR-875-5p inhibitors (**Figures 4C, D**).

### 3.5 Effects of miR-875-5p expression on cell autophagy and epithelial mesenchymal transition in cervical cancer cells

**Figure 5:**
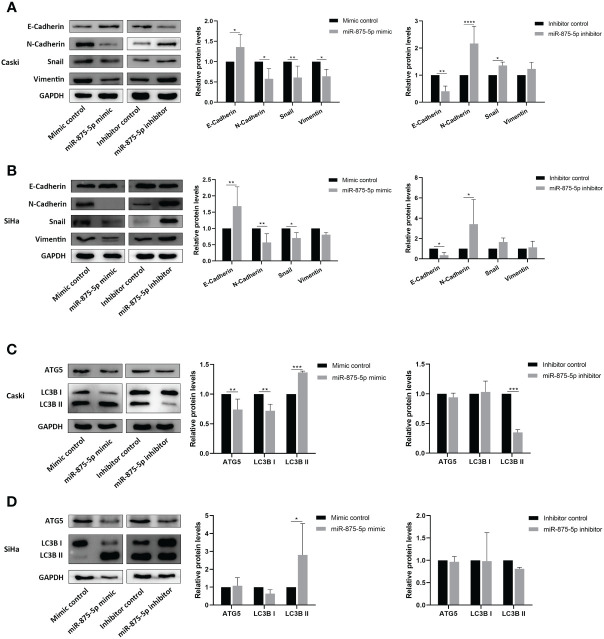
MicroRNA-875-5p inhibited the epithelial mesenchymal transition (EMT) and promoted the autophagy of CC *in vitro*. Western blot measures protein levels of **(A, B)** epithelial mesenchymal transition (EMT) related proteins E-cadherin, N-cadherin, snail and vimentin, and **(C, D)** autophagy related proteins ATG5, LC3B in CC cells *in vitro*. *p < 0.05, **p < 0.01, ***p < 0.001, ****p < 0.0001.

Western blot was used to evaluate the effects of miR-875-5p expression on EMT and autophagy in CC cells. This study screened the epithelial mesenchymal transition (EMT) related proteins, E-cadherin, N-cadherin, Snail and Vimentin, and autophagy related proteins ATG5 and LC3B as detection indicators. The results of Western blots showed that in Caski cells transfection of miR-875-5p mimic increased the protein levels of E-cadherin, and decreased the protein levels of N-cadherin, Snail, and Vimentin (**Figure 5A**). Transfection of the miR-875-5p inhibitor decreased the protein levels of E-cadherin and increased the protein levels of N-cadherin and Snail but had no significant effect on vimentin expression (**Figure 5A**). In SiHa cells, the transfection of miR-875-5p mimic increased the protein levels of E-cadherin, and decreased the protein levels of N-cadherin and Snail, but had no significant effect on Vimentin expression (**Figure 5B**). The transfection of the miR-875-5p inhibitor decreased the protein levels of E-cadherin and increased the protein levels of N-cadherin, but had no significant effect on the level of Snail and Vimentin in SiHa cells. (**Figure 5B**).

In autophagy associated proteins, the transfection of miR-875-5p mimic increased the protein levels of LC3B II and decreased the levels of ATG5 and LC3B I in Caski cells, while inhibitor transfection decreased the LC3B II level but did not affect ATG5 and LC3B I level (**Figure 5C**). In SiHa cells, miR-875-5p transfection decreased the level of LC3B II, but did not affect the level of ATG5 and LC3B I (**Figure 5D**). The transfection of miR-875-5p inhibitors did not affect ATG5, LC3B I and LC3B II level (**Figure 5C**).

### 3.6 MDM4 is the downstream target of miR−875−5p in cervical cancer

**Figure 6:**
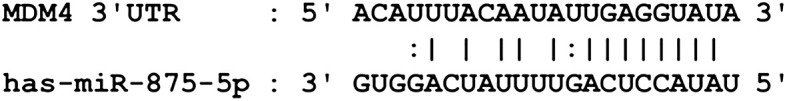
Bioinformatic analysis predicted miR−875−5p binding sites in the MDM4 mRNA 3’UTR.

Bioinformatic analysis identified that mouse double minute 4 (MDM4) is a downstream target gene for miR-875-5p and miR−875−5p binding sites in the MDM4 mRNA 3’UTR (**Figure 6**). MDM4 was confirmed as an oncogenic molecule in cervical cancer in previous studies (29, 30),.

## 4 Discussion

**Figure 7:**
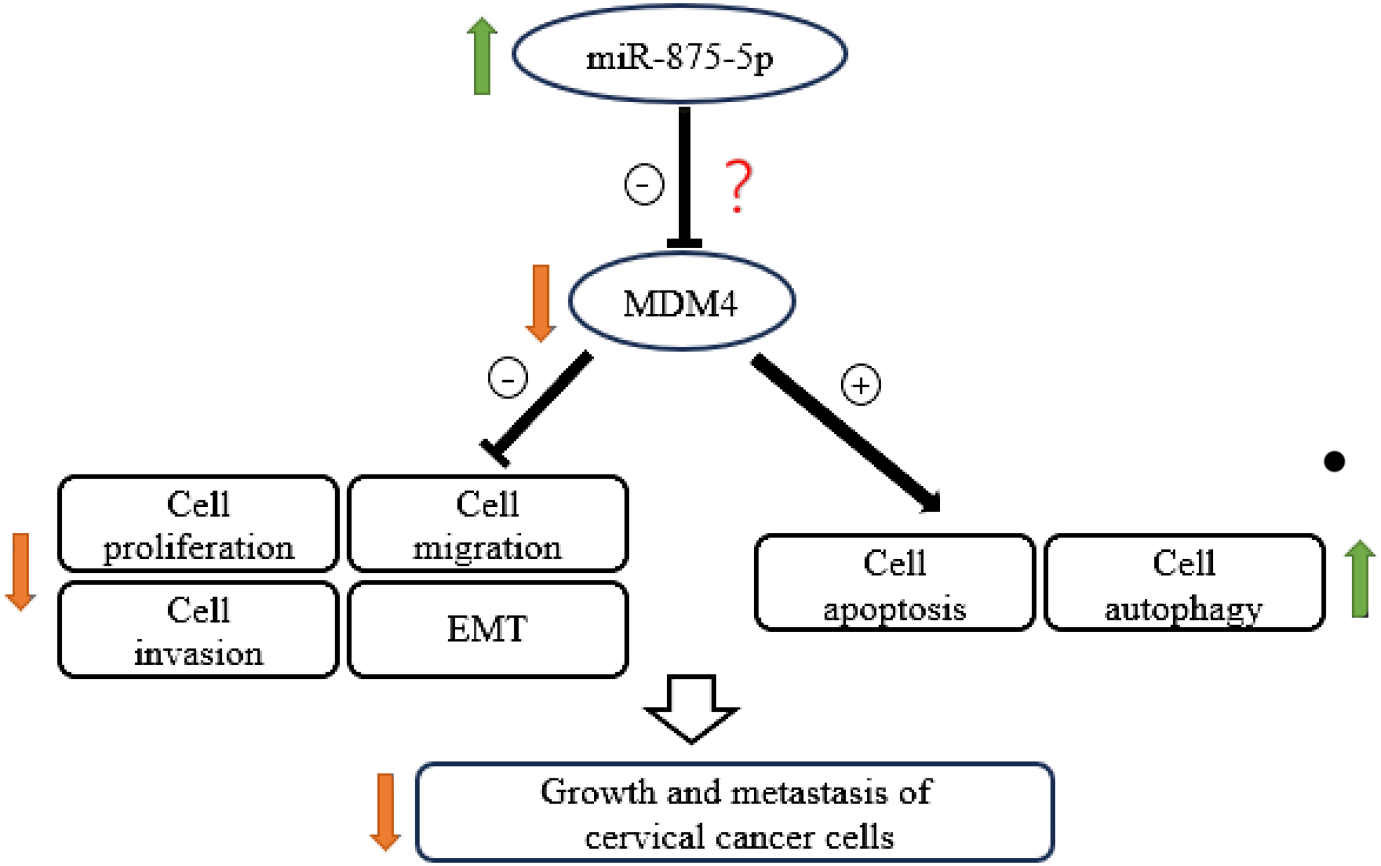
Schematic diagram showing the mechanism by which miR-875-5p overexpression inhibits the growth and metastasis of cervical cancer cells.

Cervical cancer is one of the leading causes of death among women worldwide. An accurate and economical method of screening for CC is the main challenge and would be highly beneficial for early diagnosis (31). To improve the diagnosis and treatment of CC, it is essential to explore the molecular mechanism of the occurrence and development of CC, search for biomarkers for early diagnosis/prognosis, and potential therapeutic targets. MicroRNA-875-5p affects the ability of cells to proliferate, migrate, and invade, and has been identified as a cancer-related microRNA (32, 33). However, a limited number of studies have focused on the function of miR-875-5p in CC. This study assessed the effects of miR-875-5p on CC cell lines. Our results show that miR-875-5p is dysregulated in CC cells and the expression level of miR-875-5p is related to the malignant behavior of CC cells. We found that miR-875-5p overexpression inhibits proliferation, migration, invasion, and EMT, and promotes apoptosis and autophagy in CC cells (**Figure 7**). Therefore, miR-875-5p could be a potential therapeutic target and diagnostic marker.

MicroRNAs are important post-transcriptional regulatory factors in organisms, which are widely involved in a variety of biological behaviors in cells (28, 34). A large number of studies have shown that deregulation of the expression of specific miRNAs is involved in the onset and development of different types of cancer by upregulating the expression of oncogenes (35). Hence, abnormal expression of particular miRNAs could serve as biomarkers for the screening, diagnosis, and prognosis of various types of human cancer, including CC (36, 37). Therefore, the identification of miRNAs involved in tumorigenesis could provide key clues for predicting the progression of CC and help identify new therapeutic targets. The results of this study support that a miRNA, miR-875-5p, is significantly dysregulated in CC cells compared to normal cervical epithelial cells.

As shown in **Figure 1**, miR-875-5p is significantly upregulated in C33A, Caski and MS751 cells, but downregulated in HeLa and SiHa cells. It is somewhat unexpected to observe such a large discrepancy in the level of miR-875-5p in these CC cell lines. In terms of CC types, Caski and SiHa cells were both cervical squamous carcinoma, but the expression level of miR-875-5p was up-regulated in Caski cells and down-regulated in SiHa cells. C33A and MS751 were both poorly differentiated adenocarcinoma with high expression of miR-875-5p, while HeLa is a cervical adenocarcinoma with low expression of miR-875-5p. From the perspective of HPV infection types, Caski and SiHa cells were HPV-16 infected cell lines, but their miR-875-5p expression levels were inconsistent, one was high expression and the other was low. HeLa and MS751 were HPV-18 infected cell lines, and their miR-875-5p expression levels were also inconsistent. C33A was HPV negative CC cells with high expression of miR-875-5p. From aggressiveness, miR-875-5p was highly expressed in Caski cells with slight invasion and low expressed in SiHa cells with high invasion. Other cell lines could not be evaluated due to the lack of literature assessing their invasive capacity. In summary, the expression level of miR-875-5p in CC cells did not seem to be significantly correlated with the type of HPV infection. Cervical cell types and invasion ability may affect miR-875-5p expression in CC, but due to the lack of corresponding data, the conclusion could not be made hastily and further experiments are needed to prove it. To make our research more clinically relevant, we selected two of the most commonly seen HPV16 positive cervical squamous cell lines, the Caski and SiHa cell lines, for our study. Subsequent studies have shown that low expression level of miR-875-5p is closely related to the development of CC.

The present study explored a possible role and mechanism of miR-875-5p in CC. Our results clearly demonstrated that low expression of miR-875-5p in CC cells inhibits cell apoptosis and significantly promotes cell proliferation, migration, and invasion *in vitro*. Overexpression of miR-875-5p significantly promotes apoptosis of CC cells, inhibits cell proliferation, and inhibits cell migration and invasion dependent on cell lines. These results support that miR-875-5p plays a crucial role in the growth and metastasis of CC. In agreement with our data, previous reports showed that miR-875-5p is involved in a number of types of cancer, such as gastrointestinal tract, lung and some other cancers (22, 24, 32). For example, miR-875-5p may inhibit tumor growth and metastasis in HCC by downregulating the expression of the initiation factor 3 subunit a (eIF3a) (22). Furthermore, miR-875-5p may inhibit cell proliferation in bladder cancer (24). However, the role of miR-875-5p in CC and the specific mechanism of its role remain unclear. To the best of our knowledge, various miRNAs play an important role in proliferation, metastasis, apoptosis, and development of several cancers including CC. Three of these miRNAs caught our attention, namely miR-126, miR-214 and miR-150-5p. The experiment found that miR-150-5p could regulate the expression of MDM4 and finally affect the expression of p53 gene, affect cell proliferation, migration and apoptosis in CC (29). Upregulation of miR-126 resulted in suppressed proliferation, accompanied by induced apoptosis of CC cells by down-regulating MDM4 (30). A study performed transcriptome analysis to evaluate the gene expression level of different genes after dysregulation of miR-214 which is frequently down-regulated in CC. The results demonstrated that the loss of miR-214 caused a significant upregulation of MDM4 in CC cells (38). MDM4 is a p53 binding protein that has been implicated in various cancers (38). The above MiRNA can affect the progression of CC by regulating the expression of MDM4. Although the role of MDM4 in CC has been basically confirmed in previous studies, the anticancer effect of miR-875-5p on CC has also been confirmed in this study, the relationship between miR-875-5p and MDM4 is still unclear. Based on these, we hypothesized that miR-875-5p inhibits the growth and metastasis of cervical carcinoma by targeting MDM4 (**Figure 7**). However, it cannot be ruled out that miR-875-5p can also influence other signaling pathways to exert anticancer effects. Therefore, it is necessary to investigate the expression and effect of miR-875-5p on the expression and role of MDM4 in CC.

An interesting finding is that the effect of miR-875-5p overexpression on cell migration and invasion is significantly different between Caski and SiHa cells, as shown in **Figure 3**. We don’t know why there is such a difference between these two cell lines. This might be due to the number of viral copies with carcinoma cells and the pattern in which it invades. Cervical squamous cell carcinoma cells are heterogeneous, and different cell lines and individual cells influence therapeutic outcomes. Caski cells have a higher viral copy number but are less aggressive (39). In contrast, SiHa cells have a lower viral copy number and are more invasive and aggressive (39). In terms of invasion patterns, Caski cells form large droplets of invasive cells, while SiHa cells invade as single cells or within small islands (buds) (39). Based on the differences between the two cell lines, the effect of miR-875-5p expression levels on the invasive behavior of CC cells may require further *in vitro* experiments to verify.

EMT is the process by which quiescent epithelial cells transform into mesenchymal phenotypic cells and is one of the molecular mechanisms involved in cancer cell metastasis (40). During EMT, cells lose cell-cell and cell-extracellular matrix adhesion, acquiring mesenchymal cell phenotypes. This allows the detachment of cells from the primary tumor and the separation of surrounding tissues and distant organs (41). Recent studies have shown that miR-875-5p plays an important role in influencing the EMT behavior of cancer cells (22, 42). *In vitro* experiments revealed that inhibition of miR−875−5p promotes tumor growth and metastasis by promoting HCC cell epithelial−to−mesenchymal transition progression (22). Han et al. found that linc01608 promoted EMT of HCC cells *in vitro* and *in vivo* by sponging to miR-875-5p (42). To our knowledge, our study is the first to investigate the effect of miR-875-5p on EMT in CC. Consistent with the previous reports, our data show that a high expression level of miR-875-5p inhibits EMT depending on the pathophysiology of the cell line.

An important finding is that overexpression of miR-875-5p promoted autophagy in CC cells, a novel mechanism by which miR-875-5p suppresses the growth of CC. To our knowledge, this is the first study exploring the correlation between miR-875-5p expression level and autophagy of CC cells, despite the involvement of miRs in other types of cancer (41–46). Autophagy is a highly conserved catabolic process that plays a fundamental role in maintaining cellular homeostasis (43). In the primary stages of tumor progression, it acts as an inhibitor of tumorigenesis (44). Recent studies have reported that miRNAs play a key role in autophagy of tumor cells of reproductive system tumor cells (45, 46), including CC cells (47, 48). Hu et al. found that miR-29c-3p overexpression inhibited autophagy by downregulating the forkhead box protein P1 (FOXP1)/autophagy-related gene 14 (ATG14) pathways in ovarian cancer (45). The study by Yang et al. showed that MIR-G-1 promoted serum starvation-induced nuclear autophagy and accelerated taxol (TAX)-induced DNA damage repair in CC cells (47). Exogenous expression of miR-106a greatly promoted CC cell proliferation and inhibited autophagy via targeting liver kinase B1 (LKB1) in HPV-16-associated CC (48). Consistent with previous reports, our data confirm an association between the expression level of miR-875-5p and autophagy in CC cells.

In aggregate, miR-875-5p is a newly discovered key molecule for the malignant transformation of normal cells, which may be a valuable diagnostic and prognostic marker, and a potential therapeutic target for cervical cancer. To our knowledge, no studies have evaluated the relationship between miR-875-5p and CC. Previous studies on miR-875-5p have confirmed that miR-875-5p affects cancer growth and metastasis in hepatocellular (22, 49), lung (50) and bladder (24) cancers, suggesting the potential of this miRNA as a biomarker in the diagnosis and therapeutics of cancer. The low expression of miR-875-5p has been shown to be significantly associated with an unfavorable prognosis and clinical features of HCC, including large tumor size, venous invasion and advanced TNM stage, suggesting the potential clinical importance of this miRNA (22). In the present study, our data demonstrated that the expression level of miR-875-5p was dysregulated in CC cells, suggesting that miR-875-5p could be a new potential biomarker for the CC screening. Knockdown of miR-875-5p promoted the proliferation, migration, invasion and EMT of cervical cells, along with the inhibition of apoptosis and autophagy. Overexpression of miR-875-5p achieved the opposite effects. The results confirmed that miR-875-5p served a tumor suppressive role in CC, providing a potential novel therapeutic strategy, diagnostic marker, or prognostic markers. However, this conclusion needs to be verified by clinical research. The specific mechanism may be related to the cancer-related protein MDM4, but it is not-well defined. Thus, further studies are required to determine whether MDM4 plays an anticancer role for miR-875-5p in CC.

The main limitation of our study is that the data were obtained from only *in vitro* experiments. More *in vivo* studies and studies using clinical samples are certainly needed to advance to the translational stage. Additionally, this study only evaluated the correlation between miR-875-5p and CC and did not explore how miR-875-5p regulates a particular pathway that could make sense. In the next step, more relevant studies are still needed to verify the pathway. However, we believe that the limitations are outweighed by the notable strengths and the promising future outlook of the potential new diagnostic and treatment strategy.

## 5 Conclusions

In conclusion, our data suggest that the expression of miR-875-5p in CC cells is significantly dysregulated compared to normal cervical epithelial cells. Overexpression of miR-875-5p can significantly inhibit the growth and metastasis of CC cells *in vitro*. The possible underlying mechanism may be associated with the inhibition of EMT and the promotion of apoptosis and autophagy via the MDM4 signaling pathway as shown in **Figure 7**. Our findings advance our understanding of the role and function of miR-875-5p in CC and highlight the potential value of miR-875-5p as a biomarker and promising therapeutic target for the diagnosis and treatment of CC.

## Data availability statement

The original contributions presented in the study are included in the article/supplementary material. Further inquiries can be directed to the corresponding authors.

## Ethics statement

Ethical approval was not required for the studies on humans in accordance with the local legislation and institutional requirements because only commercially available established cell lines were used.

## Author contributions

YXL: Data curation, Formal analysis, Investigation, Methodology, Resources, Software, Validation, Visualization, Writing – original draft, Writing – review & editing. CL: Data curation, Formal analysis, Investigation, Methodology, Resources, Software, Validation, Visualization, Writing – review & editing. XH: Data curation, Formal analysis, Investigation, Methodology, Software, Validation, Visualization, Writing – review & editing. YGL: Conceptualization, Formal analysis, Investigation, Methodology, Resources, Software, Supervision, Validation, Visualization, Writing – review & editing. JC: Conceptualization, Formal analysis, Funding acquisition, Investigation, Methodology, Project administration, Resources, Software, Supervision, Validation, Visualization, Writing – review & editing.
